# Microscopy analysis of the smallest subunit of the RPA complex, Rfa3p, prompts consideration of how RPA subunits gather at single-stranded DNA sites

**DOI:** 10.17912/micropub.biology.000493

**Published:** 2021-10-27

**Authors:** Agnès Ramonatxo, María Moriel-Carretero

**Affiliations:** 1 Centre de Recherche en Biologie cellulaire de Montpellier (CRBM), Université de Montpellier, Centre National de la Recherche Scientifique, 34293 Montpellier CEDEX 05, France

## Abstract

The heterotrimeric Replication Protein A (RPA) complex preserves genome integrity by protecting the single-stranded DNA that becomes exposed during repair, replication, and recombination. Its two biggest subunits, Rfa1p and Rfa2p (as named in *S. cerevisiae*) contact DNA and interact with other partners, while the smallest Rfa3p subunit is considered to fulfill a structural role. Perhaps because of this, mostly Rfa1p and eventually Rfa2p are used for microscopy studies upon tagging them with fluorophores. In this work, we explore the behavior of GFP-tagged Rfa3p basally and in response to DNA damage conditions and compare it with tagged Rfa1p. We find that fluorescent Rfa3p yields signals that are (or are detected) significantly more frequent(ly). By making a careful comparison with our own and with previously published data, we propose that Rfa3p, by virtue of its scaffolding role, may reach single-stranded DNA sites first thus serving to nucleate the full RPA complex.

**Figure 1. Fluorescence microscopy analysis of Rfa3p-GFP in untreated and in DNA damage conditions:**
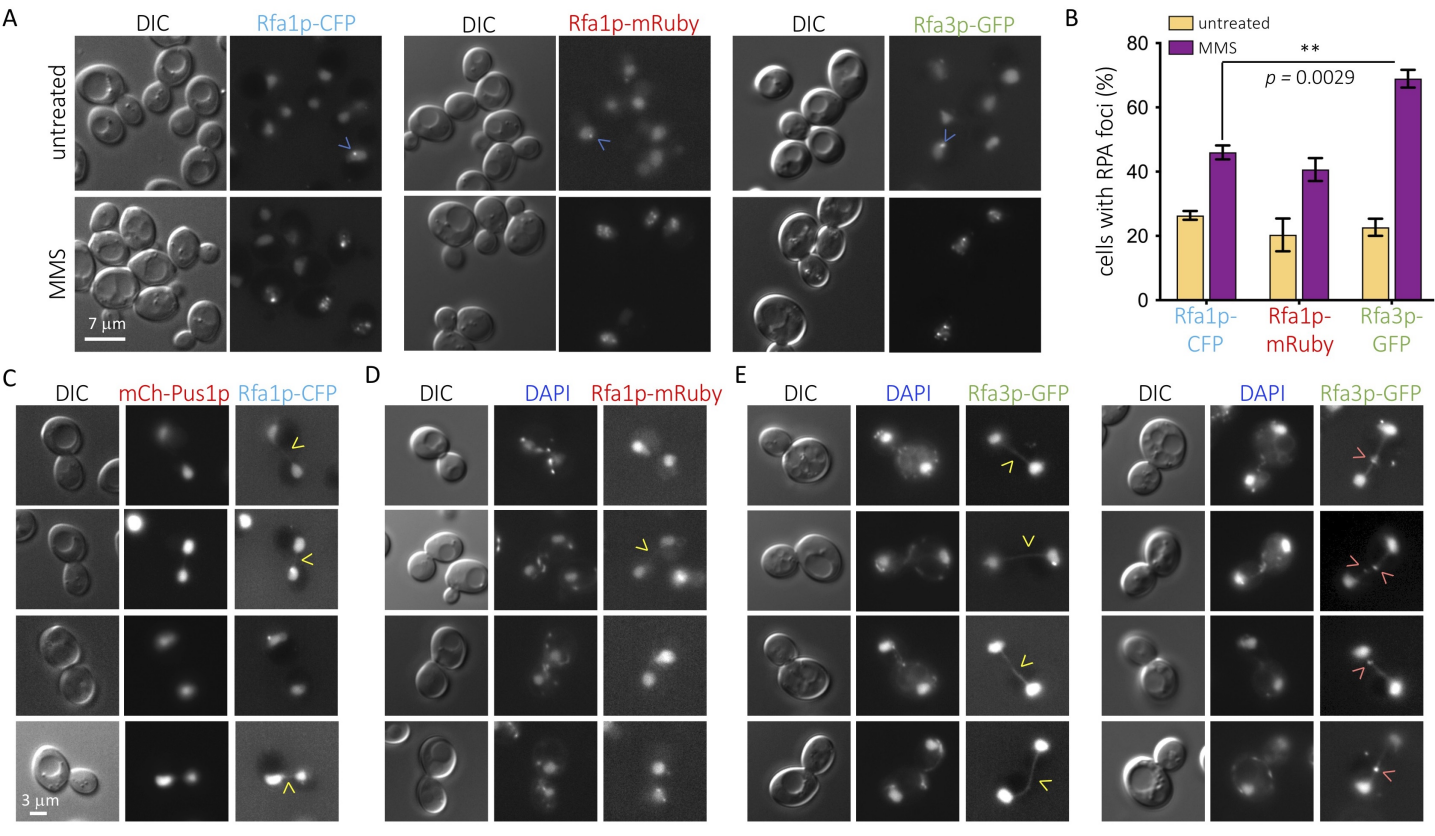
**(A)** *Saccharomyces cerevisiae* strains bearing the indicated fluorophore-tagged RPA subunits were imaged in the indicated channels after no treatment or after being exposed for three hours to 0.1% MMS. Blue arrowheads point to foci that can be observed under untreated conditions. **(B)** The percentage of cells in the population displaying at least one nuclear focus of the indicated RPA subunits was established. Each bar height indicates the mean percentage calculated out of three independent experiments, and the error bars display the standard error of that mean. The *p*-value and the asterisks indicate that the concerned means are statistically significantly different after performing a parametric *t*-test. **(C)** Four examples of mitotic cells in which the connection between mother and daughter cells’ nuclei by eventual Rfa1p-CFP signals was explored. Yellow arrowheads point at faint, rope-like Rfa1p-CFP signals that could be detected. The nucleoplasmic protein Pus1p N-terminally tagged with mCherry was used to identify the nuclei. **(D)** Same as in (C) but for Rfa1p-mRuby signals and DAPI was used to stain the DNA. Please note that mostly nuclei but also mitochondria can be seen this way. **(E) Left:** details as in (C,D), but for Rfa3p-GFP signals. Please note that the rope-like signal connecting nuclei is continuous and neat. **Right:** apart from rope-like signals connecting mother and daughter nuclei, well-defined Rfa3p-GFP foci could be detected in the connecting bridges, as pointed by pink arrowheads.

## Description

The conserved, heterotrimeric RPA complex is involved in genome stability maintenance at multiple levels. Given its affinity for single-stranded DNA, which it protects from unwanted degradation, this complex is implicated in DNA repair, replication, and recombination (Maréchal and Zou 2015). In *Saccharomyces cerevisiae*, its three subunits are called Rfa1p, Rfa2p and Rfa3p. The biggest ones, Rfa1p and Rfa2p, contact DNA directly and are also responsible for the interaction with most protein partners (for a comprehensive curation see (Maréchal and Zou 2015)). As such, their fluorophore-tagged versions have recurrently been used in microscopy studies to monitor the implication of the RPA complex in the aforementioned processes ((Lisby *et al.* 2004; Ivanova *et al.* 2020; Wong *et al.* 2020), to cite some examples). Rfa3p is the smallest subunit, with a theoretical molecular weight of 14 kDa. Its position within the 3D-structure (Yates *et al.* 2018) as well as other studies (Bochkareva *et al.* 2002) let think that Rfa3p fulfills an exclusively structural role, probably explaining why it has attracted so little interest for microscopy approaches. Only one work, to our knowledge, has simultaneously assessed the subcellular distribution of the C-terminally tagged versions of the three subunits, concluding they all mainly share a nuclear localization (Belanger *et al.* 2011). Yet, that work did not assess their potentially similar (or different) response to replication challenges or to DNA damage.

We decided to assess how the C-terminally, fluorophore-tagged versions of Rfa1p and Rfa3p compare to each other under basal conditions and in response to the alkylating agent methyl metanesulfonate (MMS). By damaging nucleotide bases, MMS provokes the accumulation of single-stranded DNA after the passage of the replication fork, thus inducing RPA participation (Wong *et al.* 2020). Not only was Rfa3p-GFP localization mainly nuclear, as reported (Belanger *et al.* 2011) but, as tagged Rfa1p, it also congregated spontaneously in the shape of foci under basal conditions (Figure 1A, upper panels, blue arrows), most likely indicative of basal DNA damage or physiological replication-associated single-stranded DNA tracts. Also as fluorescently tagged Rfa1p, which accumulates in post-replicative damaged territories in the shape of foci (Wong *et al.* 2020), Rfa3p-GFP associated in numerous foci in response to MMS (Figure 1A, bottom panels, blue arrows). Thus, we acknowledge that fluorophore-tagged Rfa3p behaves similarly to its tagged Rfa1p counterpart. Yet, when we quantified the percentage of cells displaying at least one nuclear focus, we observed that the values were reproducibly higher for the Rfa3p-tagged strain in response to MMS (Figure 1B). This was unlikely due to the type of appended fluorophore, since changing Rfa1p tag from CFP to 2 mRuby moieties did not alter the number of detected events, which always remained less abundant than when following Rfa3p (Figure 1B, asterisks), and basal values in the untreated cultures were identical for all three strains. Thus, monitoring of fluorescently tagged Rfa3p could represent a more sensitive means to study the participation, in a challenged DNA scenario, of the RPA complex.

Rfa1p and Rfa2p are also known as markers of the presence of ultra-fine bridges (UFBs) during mitosis (Germann *et al.* 2014; Liu *et al.* 2014; Ivanova *et al.* 2020). These structures mostly result from the failure to segregate unreplicated regions, either naturally difficult-to-replicate sequences or after a stress slowing down replication forks during the preceding synthesis phase. This makes them refractory to DAPI staining, as they lack chromatin features, but they are rich in single stranded DNA portions, thus prone to binding by RPA. UFBs presence in mitoses of unperturbed, cycling cells can be revealed by fluorescent Rfa1p in roughly 30 % of the cases, which manifest as weak, faint signals in the shape of a rope that would connect mother and daughter nuclei (Germann *et al.* 2014). The same events can be detected in 44 % of mitoses by using fluorescent Rfa2p yet not in the shape of a connecting rope, but of a neat focus somewhere in between both nuclei (Ivanova *et al.* 2020). We therefore explored whether Rfa3p-GFP could somehow illuminate mitosis-associated events in untreated cultures. As reported, and irrespective of the tag, Rfa1p only modestly marked bridges connecting daughter and mother cells’ nuclei during mitosis (Figure 1C,D). In sharp contrast, almost every mitosis observed in Rfa3p-GFP cells was recognizable by a neat, well-defined rope-like signal connecting both nuclei (Figure 1E, left panel). Further, in roughly half of these cases there was, additionally, one or even two defined Rfa3p foci superimposed on the rope (Figure 1E, right panel). This indicates again that fluorescent Rfa3p provides more sensitive detection than its counterparts Rfa1p or Rfa2p.

Our data have revealed that using fluorescent Rfa3p increases the sensitivity with which single stranded DNA-associated events can be detected, whether during mitosis, or in response to DNA-damaging agents. In a way, the consideration of our and other’s data from unperturbed mitoses suggest that detection sensitivity inversely correlates with the size of the tagged subunit. In this case, our data could imply that the rate of mitosis naturally concurring with at least one UFB has been underestimated. Yet, careful quantification of the time requested prior to cell division by Rfa2p foci-positive cells demonstrated these took longer than Rfa2p foci-negative ones (Ivanova *et al.* 2020), suggesting that UFBs-possessing cells can be faithfully traced with the Rfa2p-GFP tool. Alternatively, our data could mean that, despite acting as a heterotrimeric complex, the three subunits of RPA do not reach the place of action simultaneously but may assemble sequentially on-site. In this sense, and in agreement with Rfa3p fulfilling a structural role (Bochkareva *et al.* 2002; Yates *et al.* 2018), Rfa3p could reach the single stranded DNA region first and then trigger the nucleation of the full complex. This would increase its residence time at those locations, which would translate into an improved detection rate by microscopy approaches. Given the poor characterization of Rfa3p properties in the literature, it will be worth exploring this modular, on-site assembly hypothesis.

## Methods

*Saccharomyces cerevisiae* cells were grown at 25°C in rich YPD medium. All experiments were performed with exponentially growing cells. For microscopy analyses, prior to imaging, cells were incubated for 20 min with 4 µg/mL DAPI to visualize the nuclear DNA. Then, 1 mL of the culture of interest was centrifuged, the supernatant was thrown away and the pellet was resuspended in the remaining 50 μL. Next, 3 μL of this cell suspension was directly mounted on a coverslip for immediate imaging of full cells, by using the Differential Interference Contrast (DIC), and fluorescent signals by using the adequate wavelength. Imaging was achieved using a Zeiss Axioimager Z2 microscope and Metamorph software. Images were acquired at 20–23°C. Subsequent image visualization and analysis were performed with Image J v2.0.0-rc-69/1.52i. The determination of the percentage of cells displaying at least one focus per nucleus was done through visual inspection by the experimenters. GraphPad Prism was used to plot and statistically analyze the results.

## Reagents

The *RFA1*-CFP (mCherry-*PUS1::URA3*) strain (MM-03) (Lisby *et al.* 2004), *RFA1*-2xmRuby::*URA3* (MM-170) (Wong *et al.* 2020) and *RFA3*-GFP::*HIS3* (MM-286) (Huh *et al.* 2003) are W303 strains corrected for the *RAD5* gene.
